# Efficacy of Triptolide for Children with Moderately Severe Henoch-Schönlein Purpura Nephritis Presenting with Nephrotic Range Proteinuria: A Prospective and Controlled Study in China

**DOI:** 10.1155/2013/292865

**Published:** 2013-12-18

**Authors:** Li Wu, Jianhua Mao, Xia Jin, Haidong Fu, Huijun Shen, Jingjing Wang, Aimin Liu, Qiang Shu, Lizhong Du

**Affiliations:** ^1^Department of Nephrology, The Children's Hospital of Zhejiang University School of Medicine, Hangzhou, Zhejiang 310003, China; ^2^Department of Pediatrics, The Second People's Hospital of Linhai, Linhai, Zhejiang 317016, China

## Abstract

*Objective. *To observe the clinical efficacy of the Chinese herb, Triptolide, in children with moderately severe Henoch-Schönlein purpura nephritis (HSPN). *Methods*. From January 2007 to December 2011, 56 HSPN children manifested by nephrotic range proteinuria with normal kidney function and <50% crescents or sclerosing lesions on biopsy were hospitalized in the Children's Hospital of Zhejiang University School of Medicine. They were divided into two groups: the treatment group (*n* = 42; Triptolide at a dosage of 1 mg/kg*·*d, combined with prednisone at a dosage of 2 mg/kg*·*d, within a course of medium-to-long-term therapy of 6 to 9 months) and the control group (*n* = 14; prednisone alone, with the same procedure). *Results. *Short-term remission was observed in 95% of patients from treatment group and in 72% of patients from control group, respectively. There was a significant difference between both groups (*χ* ^2^ = 6.222, *P* = 0.029) for short-term effects. Meanwhile, no significant difference, as proteinuria, hematuria, hypertension, and decreased eGFR, was observed between the two groups in long-term followup (*χ* ^2^ = 3.111, *P* = 0.097). The Kaplan-Meier plot analysis also revealed no significant difference (*χ* ^2^ = 2.633, *P* = 0.105). *Conclusion.* Triptolide is effective in relieving short-term symptoms for moderately severe HSPN children, though its long-term effects need to be observed further.

## 1. Background

Henoch-Schönlein purpura (HSP) is one of the most common causes of systemic vasculitis in children. The incidence of HSP nephritis (HSPN) among HSP patients has been reported to be 15–62% [1, 2]. The long-term prognosis largely depends on renal involvement [3]. Some previous studies have described the treatment of severe HSPN with methylprednisolone and urokinase pulse therapy [4], cyclophosphamide [5, 6], cyclosporine A [7], and other medications. Even though considering the side-effects of these medications and unfavorable prognosis of the present therapy strategy in some patients, it would be worth looking for the novel and more effective treatment for the present time.

Triptolide is an effective principle of the Chinese traditional medicine tripterygium wilfordii Hook F (TwHF) that has been widely used to treat rheumatoid arthritis [8], primary glomerulonephritis, and nephrotic syndrome [9, 10] for centuries. In the present study, clinical manifestation and long-term prognosis were prospectively evaluated in children with moderately severe HSPN manifested with nephrotic range proteinuria with normal kidney function and <50% crescents or sclerosing lesions receiving Triptolide therapy.

## 2. Patients and Methods

### 2.1. Patients

From January 2007 to December 2011, 56 moderately severe HSPN patients manifested with nephrotic range proteinuria, normal kidney function, and <50% crescents or sclerosing lesions on renal biopsy were enrolled continuously. Renal biopsies were conducted at the Department of Nephrology, The Children's Hospital of Zhejiang University School of Medicine (Hangzhou city, Zhejiang province, China). All patients were graded based on glomerular pathological changes at initial biopsy [11]. Patients with ISKDC grade more than III were excluded in the present study. The clinical features, laboratory data, and prognosis were prospectively evaluated. The clinical data collection and pathological analysis were approved by the institutional ethics committee, and all of the subjects provided their written informed consent.

### 2.2. Treatment Protocol

This is a prospective controlled open-label study performed in a single center. Patients with moderately severe HSPN manifested with nephrotic range proteinuria, normal kidney function, and ISKDC grades I~III in pathology were divided into two groups.

The selection of prednisone alone or Triptolide plus prednisone was decided by the patients and/or their parents. They considered their economic situations (Triptolide is not so expensive, but it still takes some money to buy it, especially in some patients who did not have the health insurance, like those came from rural area), anxiety to side-effects (reproductive system injury, hepatic injury, etc.), and also the convenience for followup (compared with the patients receiving prednisone therapy alone, it is necessary to monitor the reproductive system and liver function in patients with combined therapy of prednisone and Triptolide).

The patients in treatment group (*n* = 42) received oral Triptolide tablets at a dosage of 1 mg/kg*·*d (with a daily dose of less than 60 mg and a course of 3 to 6 months) and prednisone at a dosage of 2 mg/kg*·*d (maximum daily dose 60 mg) and maintained this dose for 4 weeks. After that, prednisone was tapered off gradually within 6 to 9 months.

The patients in control group (*n* = 14) received prednisone alone with the same procedure as mentioned from patients in treatment group but without Triptolide.

Patients in both groups received ACEi and supportive therapies.

### 2.3. Histopathology

Biopsies were performed before treatment was administered. All specimens were examined by light microscopy, immunofluorescence microscopy, and electron microscopy. A biopsy slice containing eight or more glomeruli was considered adequate for histological analysis. The biopsy findings were graded according to the ISKDC classification [11]: grade I: minimal glomerular abnormalities; grade II: mesangial proliferation without crescents or sclerosing lesions; grade III: focal segmental (IIIa) or diffuse (IIIb) mesangial proliferation with <50% crescents or sclerosing lesions; grade IV: mesangial proliferation with 50–70% crescents or sclerosing lesions; grade V: mesangial proliferation with >75% crescents or sclerosing lesions; grade VI: membranoproliferative-like lesions. Patients with ISKDC grades IV~VI were excluded the in present study.

### 2.4. Definitions for Short-Term Response [12]

Complete remission was defined as clinical symptoms and signs disappeared and urine protein was negative for three consecutive measurements or proteinuria was less than 4 mg/h per m^2^ body surface area.

Partial remission was diagnosed if proteinuria was reduced to 4.1–40 mg/h per m^2^ body surface area, with an increase of serum albumin concentration to >35 g/L within 6 months.

A nonresponsive patient was diagnosed if there was no improvement in clinical symptoms or signs 6 months after the therapy of Triptolide with or without prednisone, urinary protein remained as +++ or greater or proteinuria was more than 40 mg/h per m^2^ body surface area, and serum albumin concentration was <35 g/L.

### 2.5. Definitions for Long-Term Prognosis

The long-term outcomes were classified as (A) normal and minor urinary abnormalities (including healthy patients without urinary abnormalities and those with microalbuminuria (UA/C 2.5–25 mg/mmol); (B) persistent mild proteinuria and GFR ≥90 mL/min/1.73 m^2^ (two standard deviations below the mean value of controls) with or without hematuria; (C) active renal disease (hypertension with mean arterial pressure (MAP) >95th percentile and/or UA/C ≥200 mg/mmol and/or GFR 60–90 mL/min/1.73 m^2^) with or without hematuria; (D) chronic renal failure (GFR ≤60 mL/min/1.73 m^2^) or end-stage renal disease (ESRD, requiring dialysis and/or renal transplantation). Categories A and B were considered good outcomes and categories C and D poor outcomes.

### 2.6. Statistical Analysis

SPSS (version 18.0 for windows) was used for all statistical analyses. Correlations were evaluated by the Chi-square test. $\overset{-}{X}\pm \text{S}$ was representative of measurement data. Student's *t-*test was used for comparison of enumeration data between groups. *P* < 0.05 referred to a statistically significant difference.

## 3. Results

### 3.1. Baseline Patient Characteristics

Clinical and biochemical characteristics of the 56 patients recruited in present study are summarized in Table 1. The baseline clinical and laboratory characteristics were similar between two groups, and no significant difference was found in all indices (gender, age, course, pathological type, urine protein quantification, serum albumin concentration, and serum creatinine (Scr)) between both groups (Table 1).

### 3.2. Short-Term Response to Therapy

We evaluated the short-term response to Triptolide with or without prednisone therapy according to the outcomes of patients within 6-month interval. The results were summarized in Table 2. There is a significant difference on short-term response between treatment and control groups (40/42 versus 10/14, *χ* ^2^ = 6.222, and *P* = 0.029).

### 3.3. Long-Term Prognosis in Two Groups

We evaluated the long-term prognosis to Triptolide with or without prednisone therapy according to the outcomes of patients after 6.1~70 months of followup. The results were summarized in Table 3. No significant difference was found between two groups on long-term prognosis after 6.1~70 months of followup (*χ* ^2^ = 3.111, *P* = 0.097).

**Figure 1:**
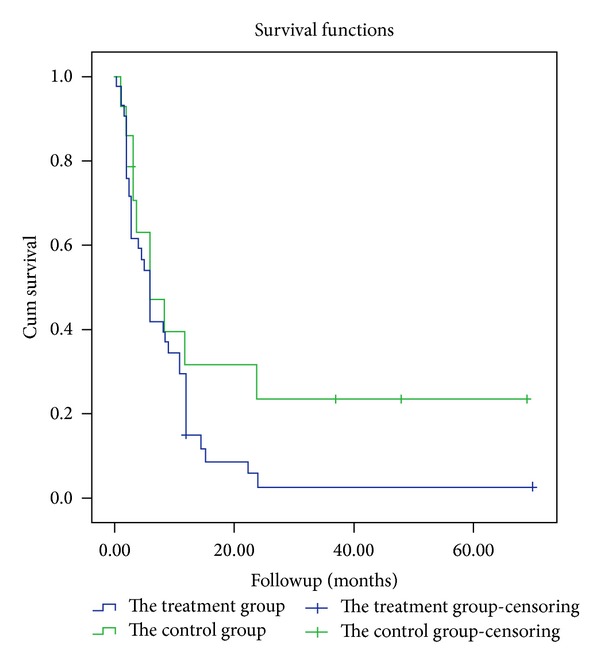
Survival analysis for analyzing the difference of long-term prognosis between control and treatment groups by Kaplan-Meier method. The result indicated no significant difference, *χ* ^2^ = 2.633 and *P* = 0.105.

Survival analysis by Kaplan-Meier method was used to analyze the difference of long-term prognosis between two groups, which also further indicated no significant difference, *χ* ^2^ = 2.633, *P* = 0.105 (Figure 1).

One case in Triprolide group and one case in control group presented a poor prognosis switched to the mycophenolate mofetil (MMF) therapy. Proteinuria and hematuria were ameliorated in both patients after MMF therapy, though no significant improvements were seen in eGFR from both patients.

Two patients from Triprolide group received repeated renal biopsy. The first one is an 8-year-old girl with ISKDC grade IIb from first renal biopsy. She presented with ISKDC grade IIa at secondary renal biopsy after 2.5-year followup. The second one is a 6-year-old boy with ISKDC grade IIa from first renal biopsy. He presented with ISKDC grade IIIb at secondary renal biopsy after 4.5-year interval.

### 3.4. Adverse-Effects

Possible side effects induced by Triptolide and/or steroids treatment were listed in Table 4, except for some side-effects only related to prednisone, such as Cushingoid changes. No severe or long-term side-effects associated with prednisone were developed, such as osteoporosis, peptic ulcer, electrolyte disturbance, hypertension, myasthenia, amyotrophy, myositis, ocular hypertension, or hypofunction of sebaceous glands.

## 4. Discussion

Renal involvement is the principal cause of morbidity and mortality in children with HSP, and according to the literature, in some specialized centers, the proportion of children with HSPN that progressed to renal failure or end-stage renal disease varies from 1 to 17%. Thus, it is important to ascertain the most appropriate treatment for patients with HSPN [13].

A number of previous studies have described the treatment of severe HSPN with methylprednisolone and urokinase pulse therapy [4], cyclophosphamide [5, 6], cyclosporine A [7], mycophenolate mofetil (MMF) [14, 15], and other medications. Park et al. [16] retrospectively analyzed 29 cyclosporine A-treated HSPN patients with nephrotic range proteinuria and ISKDC grades I to IIIb and demonstrated that cyclosporine A (CsA) is a very effective and safe method, although some patients became cyclosporine A dependent. Jauhola et al. [17] compared CsA and methylprednisolone pulses (MP) therapy in the treatment of HSPN children with nephrotic-range proteinuria or ISKDC grades II to III. Their results demonstrated that all CsA-treated patients achieved resolution of nephrotic-range proteinuria within 3 months, while the MP group response was slower and in 6/13 was not achieved with the initial treatment, indicating that treatment of HSPN with CsA is efficacious, safe, and not inferior to MP. Du et al. [14] reported that MMF was used for treatment of 12 HSPN children with nephrotic-range proteinuria and ISKDC grades II to IV, and the results revealed that all patients responded to MMF at a mean of 2.5 months and at last followup (mean followup 3.9 years), all patients had negative proteinuria and normal renal function, and no relapses were noted. They concluded that MMF is also useful for treating pediatric patients with HSPN and nephrotic-range proteinuria.

In 2012, New KDIGO (Kidney Disease: Improving Global Outcomes) has published clinical practice guidelines on various glomerulonephritides including HSPN using evidence-based principles [18]. Even so considering the fact that there are no data, other than small observational studies, examining the treatment of moderately severe HSPN patients with nephrotic range proteinuria, normal kidney function, and <50% crescents or sclerosing lesions on renal biopsy, there is a call for RCT study comparing a 6- to 12-month course of corticosteroids to shorter-duration corticosteroids.

Triptolide is an effective principle of the Chinese Traditional Medicine Tripterygium wilfordii Hook F (TwHF) that has been widely used to treat autoimmune and inflammatory diseases such as rheumatoid arthritis [8], IgA nephropathy [10], and nephrotic syndrome [9, 19] for centuries. Recently a meta-analysis of Triptolide for idiopathic refractory nephrotic syndrome in adults has been accomplished [19]. It was found that Triptolide indeed resulted in a significant increase of complete remission (odds ratio (OR) 2.81, 95% CI 1.26 to 6.30) and total remission (OR 3.25, 95% CI 1.20 to 8.76) compared with cyclophosphamide or placebo. Similar meta-analysis of Triptolide for IgA nephropathy has been conducted also [10]. The results revealed that Triptolide brought about a favorable increase in complete remission (RR 1.53, 95% CI 1.09 to 2.16, *I* ^2^ = 12%) and total remission (RR 1.27, 95% CI 1.08 to 1.48, *I* ^2^ = 0%) compared with non-Triptolide treatment, demonstrating that Triptolide was certainly a valuable and promising immunosuppressive remedy for IgA nephropathy.

In our previous study [20], we evaluated the clinical features and prognosis of 101 children with HSPN, ISKDC grades IIIa/IIIb from January 1992 to November 2008. Our study revealed that no significant difference in clinical manifestations and long-term prognosis of HSPN patients with grade IIIa or grade IIIb was found, and for grade IIIa or IIIb HSPN patients, whether or not accompanied with nephrotic range proteinuria, Triptolide alone is enough, and combination therapy with steroid did not improve their clinical outcomes eventually.

Based on these findings, the primary aim of present study was to determine the effect of Triptolide in treatment of moderately severe HSPN patients manifested with nephrotic range proteinuria, normal kidney function, and <50% crescents or sclerosing lesions on renal biopsy. The present study demonstrated that Triptolide is effective to relieve short-term symptoms for these patients, 95% (40/42) patients presented short-term response in Triptolide-treated group compared with 72% (10/14) patients manifested with short-term response in control group. Even though there are no significant differences in long-term prognosis between the two groups after 6.1~70 months of followup, its long-term effects in moderately severe HSPN patients need to be observed further.

Above all, this is a consecutive study evaluating the effects of Triptolide for moderately severe HSPN patients manifested with nephrotic range proteinuria, normal kidney function, and <50% crescents or sclerosing lesions on pathology. Combined with basic treatment of steroids, additional Triptolide administration might be effective to relieve short-term symptoms, though its long-term effects need to be evaluated for more patients in future.

## Figures and Tables

**Table 1:** Baseline patient characteristics in 56 children with moderately severe Henoch-Schönlein purpura nephritis.

	Treatment	Control	*P* value
Number of cases	42	14	

Boy/girl	24/18	5/9	0.165
Age (year-old)	8.26 ± 2.87	8.87 ± 2.59	0.479
Course (days)	13.62 ± 12.147	18.71 ± 16.754	0.308
ISKDC pathological type			
Grade I	0 (0%)	2 (14.3%)	0.089
Grade II	31 (73.8%)	9 (64.3%)	
Grade III	11 (26.2%)	3 (21.4%)	
24 hr proteinuria (mg)	2290.63 ± 1313.90	2362.69 ± 1083.76	0.840
Serum albumin (g/L)	33.43 ± 6.17	36.51 ± 5.49	0.136
Scr (*μ*mol/L)	38.68 ± 12.02	33.58 ± 8.99	0.131

**Table 2:** The short-term response in different groups with moderately severe Henoch-Schönlein purpura nephritis.

Group	Cases	Complete remission	Partial remission	No response	Total remission rate
Treatment	42	14 (33%)	26 (62%)	2 (5%)	95%
Control	14	5 (36%)	5 (36%)	4 (28%)	72%

*χ* ^2^ = 6.222, *P* = 0.029.

**Table 3:** Long-term prognosis in different groups with moderately severe Henoch-Schönlein purpura nephritis.

Group	Cases	Follow-up times (months)	A	B	C	D
Treatment	42	40.76 ± 19.18	29 (69%)	9 (21%)	2 (5%)	2 (5%)
Control	14	38.14 ± 22.51	8 (57.1%)	2 (14.3%)	2 (14.3%)	2 (14.3%)

*χ* ^2^ = 3.111, *P* = 0.097. (A) Normal and minor urinary abnormalities (including healthy patients without urinary abnormalities and those with microalbuminuria (UA/C 2.5–25 mg/mmol) with or without hematuria); (B) persistent mild proteinuria and GFR ≥90 mL/min/1.73 m^2^ (two standard deviations below the mean value of controls) with or without hematuria; (C) active renal disease (hypertension with mean arterial pressure (MAP) >95th percentile and/or UA/C ≥200 mg/mmol and/or GFR 60–90 mL/min/1.73 m^2^) with or without hematuria; (D) chronic renal failure (CRF) (GFR ≤60 mL/min/1.73 m^2^) or end-stage renal disease (ESRD, requiring dialysis and/or renal transplantation).

**Table 4:** Reported side effects in 56 children with moderately severe Henoch-Schönlein purpura nephritis treated as Triptolide with or without prednisone.

	Treatment group	Control group	*P* value
*n*	42	14	

Liver injury	5	1	0.618
Hypertension	2	0	0.559
Hyperglycemia	1	1	0.441
Gastrointestinal reaction	2	0	0.559
Leukocytopenia	4	0	0.305
